# Author Correction: Apoptotic stress causes mtDNA release during senescence and drives the SASP

**DOI:** 10.1038/s41586-023-07002-7

**Published:** 2024-01-02

**Authors:** Stella Victorelli, Hanna Salmonowicz, James Chapman, Helene Martini, Maria Grazia Vizioli, Joel S. Riley, Catherine Cloix, Ella Hall-Younger, Jair Machado Espindola-Netto, Diana Jurk, Anthony B. Lagnado, Lilian Sales Gomez, Joshua N. Farr, Dominik Saul, Rebecca Reed, George Kelly, Madeline Eppard, Laura C. Greaves, Zhixun Dou, Nicholas Pirius, Karolina Szczepanowska, Rebecca A. Porritt, Huijie Huang, Timothy Y. Huang, Derek A. Mann, Claudio Akio Masuda, Sundeep Khosla, Haiming Dai, Scott H. Kaufmann, Emmanouil Zacharioudakis, Evripidis Gavathiotis, Nathan K. LeBrasseur, Xue Lei, Alva G. Sainz, Viktor I. Korolchuk, Peter D. Adams, Gerald S. Shadel, Stephen W. G. Tait, João F. Passos

**Affiliations:** 1 Department of Physiology and Biomedical Engineering, Mayo Clinic, Rochester, MN USA; 2 Robert and Arlene Kogod Center on Aging, Mayo Clinic, Rochester, MN USA; 3 Biosciences Institute, Faculty of Medical Sciences, Campus for Ageing and Vitality, Newcastle University, Newcastle upon Tyne, UK; 4 ReMedy International Research Agenda Unit, IMol Polish Academy of Sciences, Warsaw, Poland; 5 Cancer Research UK Scotland Institute, Glasgow, UK; 6 School of Cancer Sciences, University of Glasgow, Glasgow, UK; 7 Institute of Developmental Immunology, Biocenter, Medical University of Innsbruck, Innsbruck, Austria; 8 Wellcome Centre for Mitochondrial Research, Biosciences Institute, Newcastle University, Newcastle upon Tyne, UK; 9 Center for Regenerative Medicine, Department of Medicine, Massachusetts General Hospital, Boston, MA USA; 10 Harvard Stem Cell Institute, Harvard University, Cambridge, MA USA; 11 Sanford Burnham Prebys Medical Discovery Institute, La Jolla, CA USA; 12 Degenerative Diseases Program, Sanford Burnham Prebys Medical Discovery Institute, La Jolla, CA USA; 13 Newcastle Fibrosis Research Group, Biosciences Institute, Newcastle University, Newcastle upon Tyne, UK; 14 Department of Gastroenterology and Hepatology, School of Medicine, Koç University, Istanbul, Turkey; 15 Instituto de Bioquímica Médica Leopoldo de Meis, Universidade Federal do Rio de Janeiro, Rio de Janeiro, Brazil; 16 Division of Oncology Research and Department of Molecular Pharmacology and Experimental Therapeutics, Mayo Clinic, Rochester, MN USA; 17 Department of Biochemistry, Department of Medicine, Montefiore Einstein Cancer Center, Wilf Family Cardiovascular Research Institute, Institute for Aging Research, Albert Einstein College of Medicine, New York, NY USA; 18 Cancer Genome and Epigenetics Program, Sanford Burnham Prebys Medical Discovery Institute, La Jolla, CA USA; 19 Salk Institute for Biological Studies, La Jolla, CA USA; 20 Department of Pathology, Yale University School of Medicine, New Haven, CT USA

**Keywords:** Senescence, Preclinical research

Correction to: *Nature* 10.1038/s41586-023-06621-4 Published online 11 October 2023

In the version of this article initially published, US NIH grants R33AG61456-4 and R01AG064165 were missing from the Acknowledgements section and have now been inserted in the HTML and PDF versions of the article.

