# Supplementary material for: Apoptotic stress causes mtDNA release during senescence and drives the SASP
Source: Nature. 2023 Oct 11;622(7983):627–36. doi: 10.1038/s41586-023-06621-4 (PMC10584674; doi:10.1038/s41586-023-06621-4)
Supplement: Supplementary file 1 — Supplementary Figs. 1–9 and Supplementary Tables 1–4. [file 41586_2023_6621_MOESM1_ESM.pdf]

nature portfolio

https://doi.org/10.1038/s41586-023-06621-4

Supplementary information

Apoptotic stress causes mtDNA release during senescence and drives the SASP

In the format provided by the authors and unedited

Supplementary Figure 1 uncropped western blots

Figures 1d & 2g

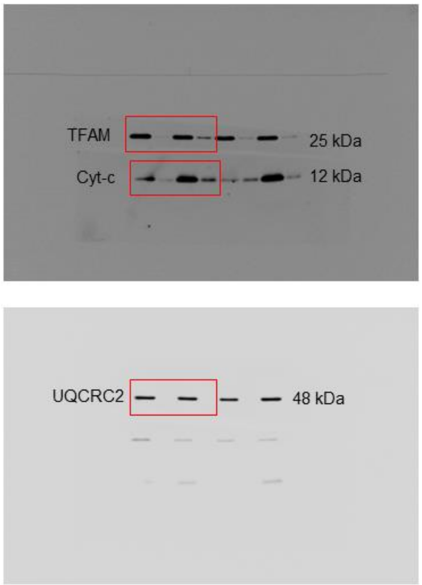

Figure 1e

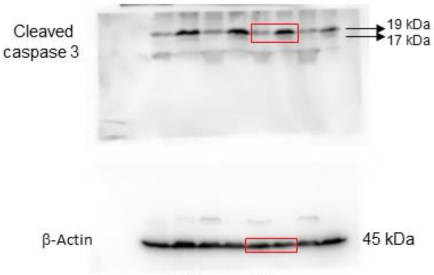

Figures 1f

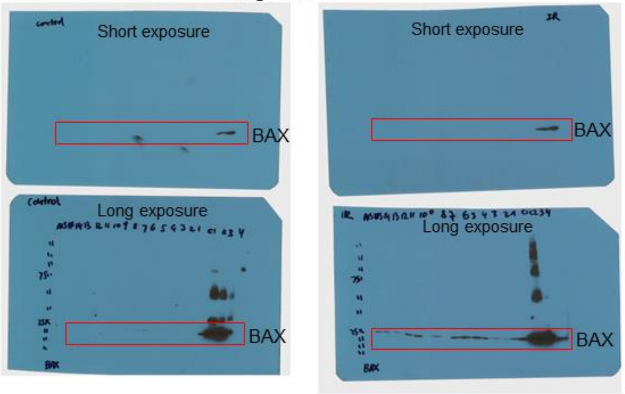

Figure 3a

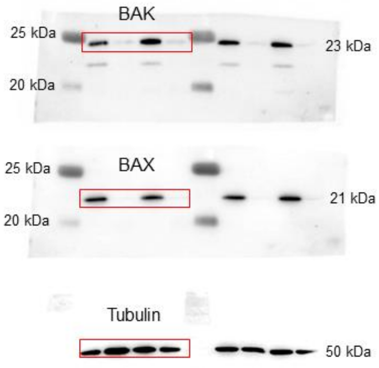

Figure 3j

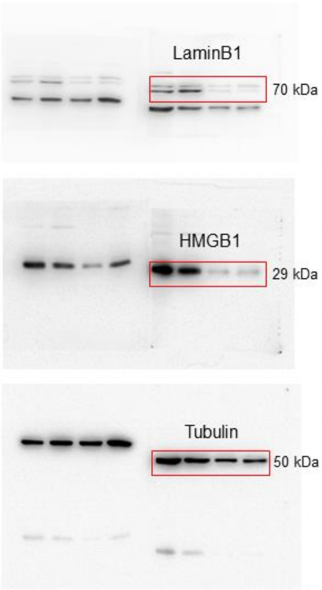

Figure 5b

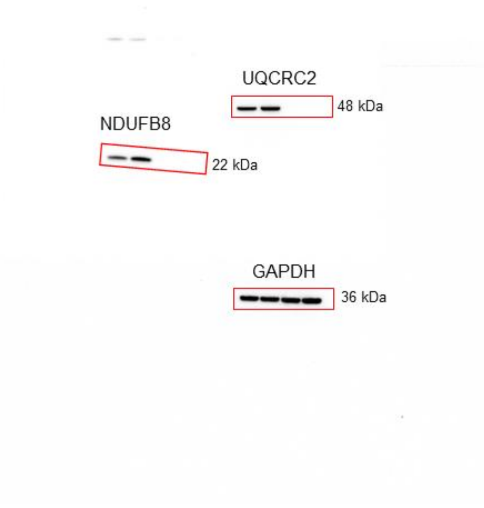

Extended Data Figure 1a

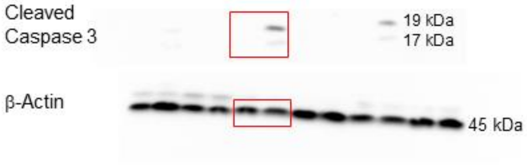

Extended Data Figure 4b

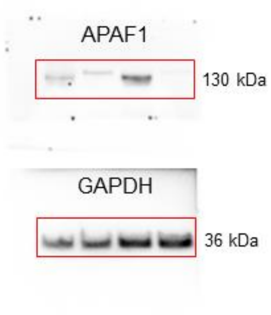

Extended Data Figure 7c

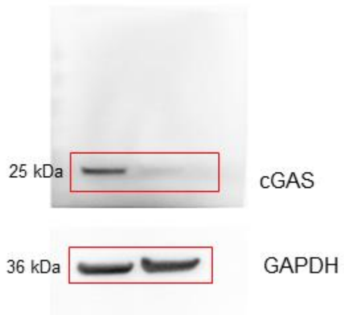

Extended Data Figure 8c

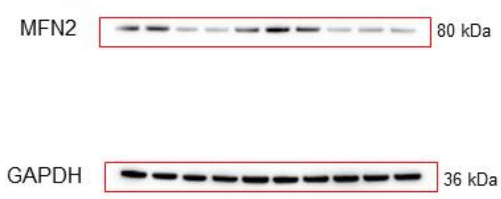

Extended Data Figure 9b

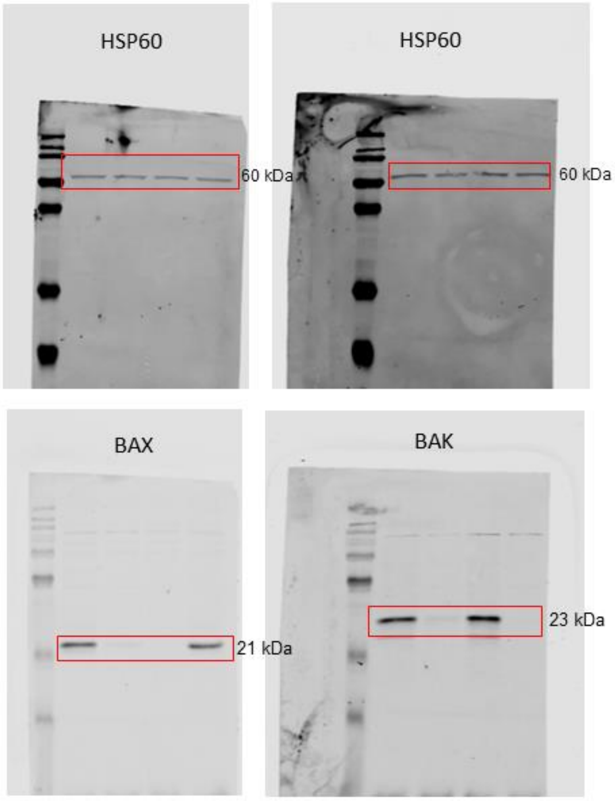

Extended Data Figure 10f

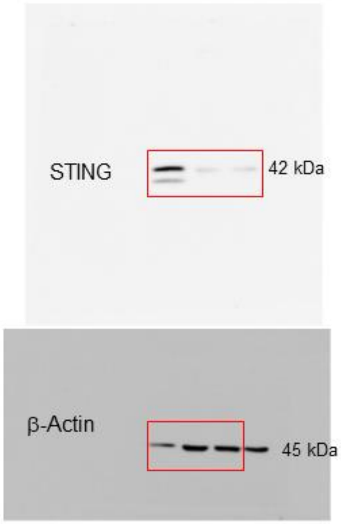

Supplementary Figure 2

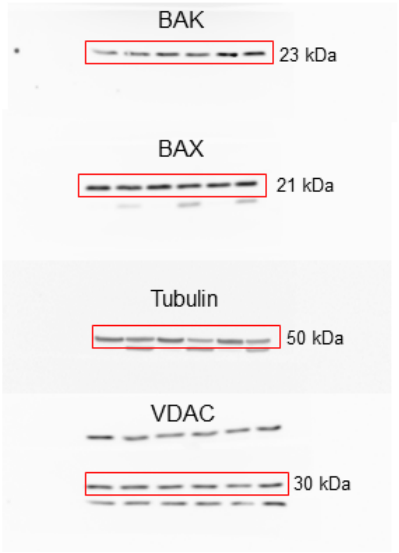

Supplementary Figure 7c

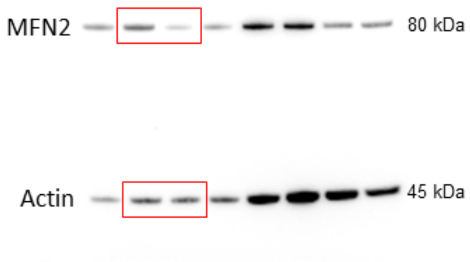

Supplementary Figure 1- Source data for western blot analysis. Uncropped Western blot raw data. The molecular weight marker is indicated on the left (given in kDa). Boxes with red lines indicate cropped regions as presented in the main, extended data and supplementary figures.

Supplementary Figure 2

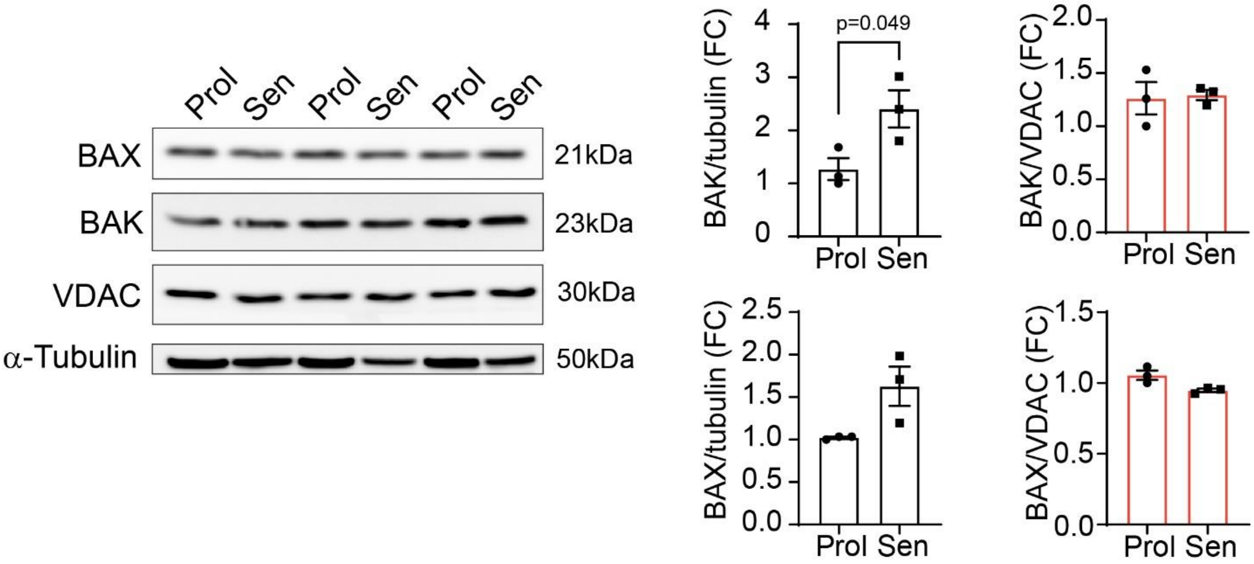

Supplementary Figure 2 – Expression of BAX and BAK in proliferating and senescent MRC5 fibroblasts.

(Left) Western blotting showing expression of BAX, BAK, VDAC and α-tubulin in proliferating and senescent (IR) MRC5 fibroblasts. (Right) Quantification of expression of BAX and BAK normalized either to α-tubulin or VDAC. Data are mean of n=3 independent experiments ± S.E.M. Statistical significance (two-sided Student's unpaired t-test) is indicated. For gel source data, see Supplementary Figure 1.

Supplementary Figure 3

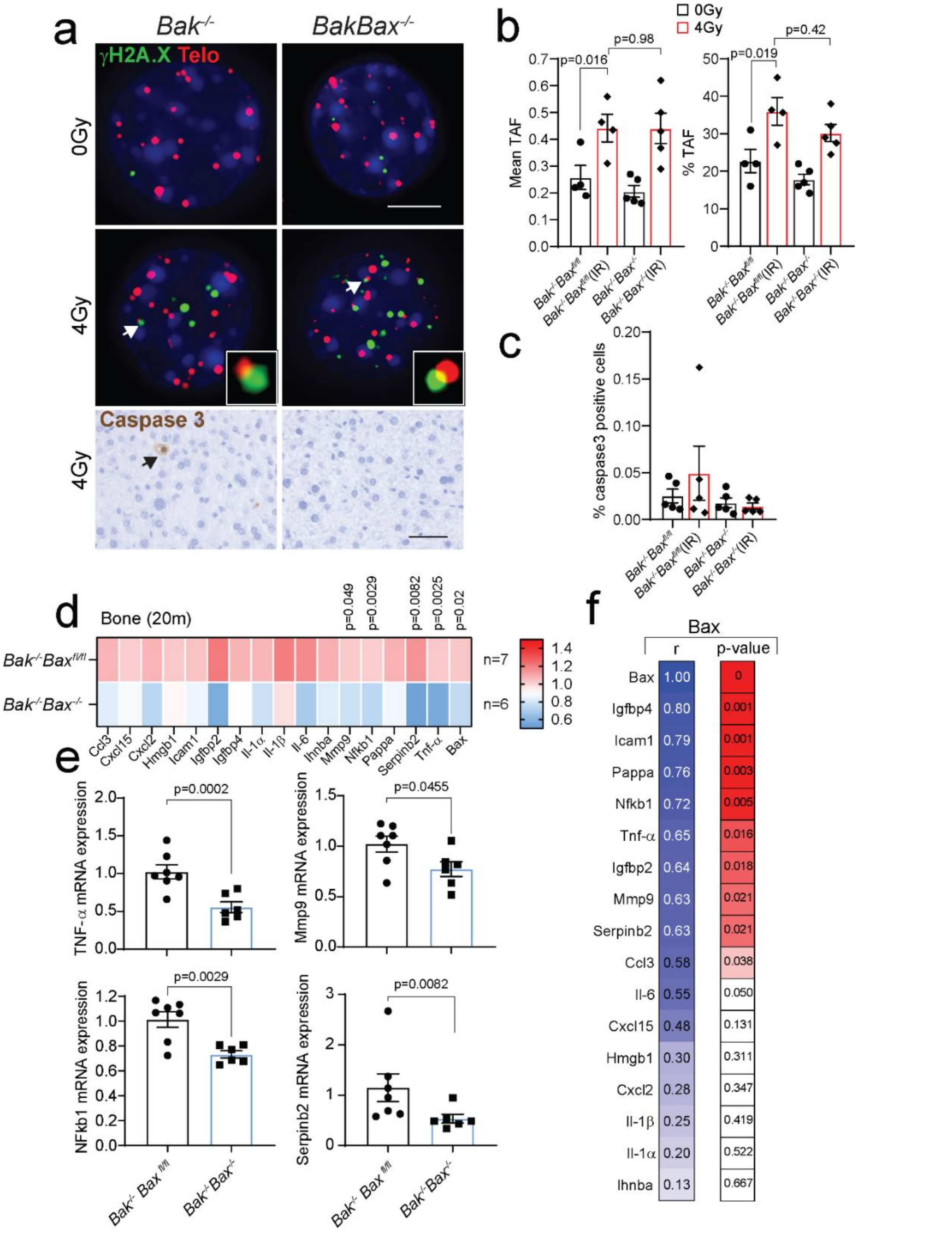

Supplementary Figure 3 – Effect of AAV-Cre-mediated deletion of BAX and BAK in aged liver and bone.

(a) (Top) Representative immuno-FISH image in liver sections from Sham and 4Gy-irradiated *Baxfl/fl Bak-/-* and *BakBax-/-* mice. γH2A.X is shown in green, telomeres are in red, and colocalization between the two is indicated by arrows and amplified at the bottom (scale bar is

5μm). (Bottom) Representative immunohistochemical image of caspase-3 in liver sections from irradiated *Baxfl/fl Bak-/-* and *BakBax-/-* mice (scale bar is 80μm). Quantification of **(b)** mean number of Telomere-associated foci (TAF) (left) and percentage of hepatocytes containing TAF (right) (n=4 Sham-IR and 4Gy-IR $Bak^{-/-}$ $Bax^{fl/fl}$; n=5 Sham-IR and 4Gy-IR $Bak^{-/-}$ $Bax^{-/-}$ and **(c)** percentage of cells positive for caspase-3 in livers from *Baxfl/fl Bak-/-* and *BakBax-/-* mice (n=5) in the conditions indicated. **(d)** Heatmap showing mRNA levels of SASP factors and of Bax in the femur from aged *Baxfl/fl Bak-/-* following tail vein injection of AAV-Cre virus. **(e)** Graphs showing quantification of genes significantly reduced in **(d)**. **(f)** Heatmap showing correlation coefficients between expression levels of Bax and different SASP factors in the bone of aged $Bax^{fl/fl}$ $Bak^{-/-}$ (n=7) and $BaxBak^{-/-}$ (n=6) mice. Data are mean ± S.E.M. Statistical significance was assessed using one-way ANOVA followed by Tukey's multiple comparison test (b, c), two-sided Student's unpaired t-test and Mann Whitney test (d and e).

Supplementary Figure 4

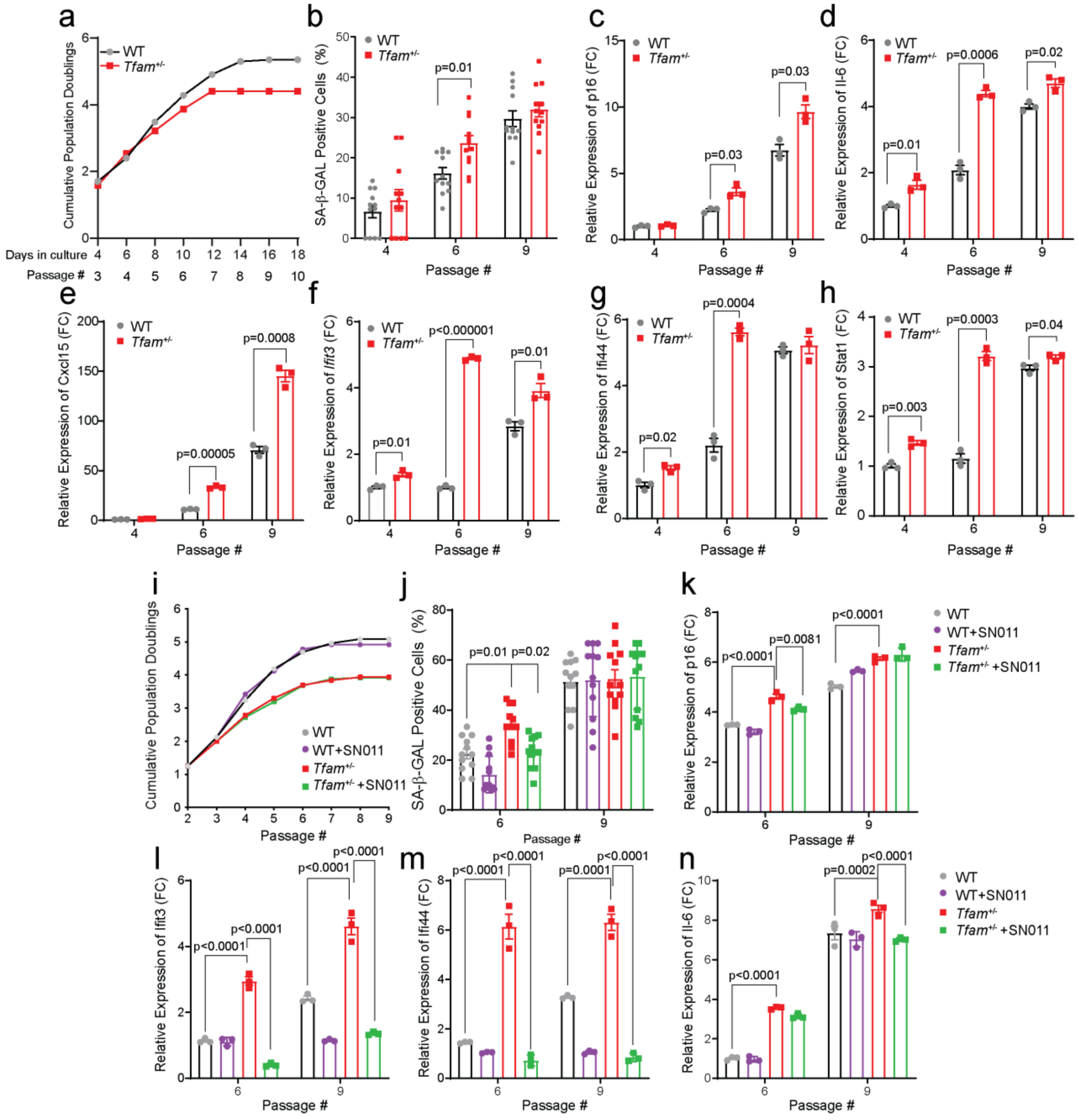

Supplementary figure 4 – *Tfam+/-* MEFs show early onset of replicative senescence and increased SASP.

(a) Cumulative population doublings of Mouse embryonic fibroblasts (MEFs) derived from wildtype (WT) and *Tfam+/-* mice during 10 serial passages. Senescence was confirmed by **(b)** quantification of SA-β-GAL positive cells and **(c)** RT-qPCR assessment of p16Ink4a expression, as well as **(d,e)** RTqPCR assessment of SASP genes (Il-6 and Cxcl15) relative to Actin levels. **(f -h)** RT-qPCR assessment of ISGs (Ifit3, Ifi44, and Stat1) relative to Actin levels. **(i)** Cumulative

population doublings of MEFs derived from WT and *Tfam+/-* mice treated with vehicle (UNT) or 0.5uM of STING inhibitor SN011 during 9 serial passages. Senescence was confirmed by **(j)** quantification of SA-β-GAL positive cells (12 images *per* condition from 2 biological replicates; error bars indicate mean ± S.E.M) and **(k)** RT-qPCR assessment of p16Ink4a expression relative to Actin levels at passages 6 and 9. **(l-m)** RT-qPCR assessment of ISGs (Ifit3 and Ifi44) and **(n)** Il-6 relative to Actin levels at passages 6 and 9. Data are mean ± S.E.M of n=3 independent experiments. Statistical significance was assessed by Two-sided unpaired t-test followed by Holm-Sidak's multiple comparison test (b-h) and One-Way ANOVA followed by Tukey's multiple comparison test (j-n).

Supplementary Figure 5

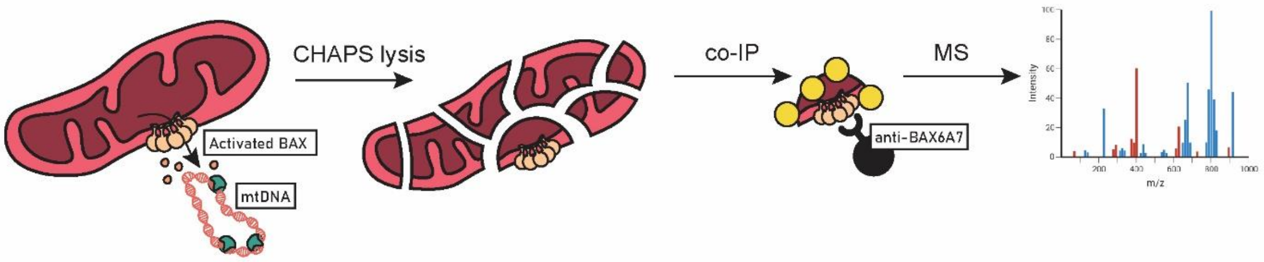

MS proteomic analysis of activated-BAX interactome in senescent cells.

biological process (GO term-based)	interactors of activated BAX
mitochondrial function	mitochondrial dynamics and trafficking:<br>MFN2, ARMCX1, ARMCX2, VAT1<br>mitochondrial energy metabolism:<br>NDUFV2, NDUFB2, NDUFB11, NDUFC2, EFTB, SMIM20, DLAT<br>mitochondrial lipid homeostasis:<br>NME4, ACOT2<br>other functions:<br>DNAJA3, FAM210B, MRPS34, ATAD1,
immunity	CDC42SE1, PLGRKT, DEFA3, S100A7, LTA4H, LRCH4, IGKC, HLA-C, LYZ, IGLL5
cytoskeleton organization	SYNE2, GSN, CDC42EP3, ACTN4, SNTB2, FSCN1, AFAP1, CAP1, TUBGCP2, TUBB6
protein folding, degradation and transport	FBXL20, SMURF1, MAN1B1, CAPN1, DNAJA2, STIP1, FKBP15, FKBP7, DNAJB2 DNAJB11, SUMF1, AP1S1, CEP41, SEC31A, KDELR3, TNPO1
other processes	nucleic acids metabolism:<br>RBM33, UPF1, MBNL2, NCOR1, YBX3, ZNF445, SAP130<br>cell fate and apoptosis:<br>DNAJB4, STEAP3, DNAJA3<br>cytoplasmic vesicles:<br>EXOC4, VTI1A, VPS11<br>other:<br>SLC39A10, ANTXR1, CDSN, DSG1, DSC1, PPFIBP1, FBN2, ATP6V1D, EIF3G, EIF4A2, ZC3H15, CALML5, ANKS1B, TMEM256, KPRP, TMEM57, PRR14L

Supplementary Figure 5 – Activated-BAX interactome includes mitochondrial dynamics factors.

(**a**) Schematic representation of the experimental design aimed at identification of activated-BAX interactors. Anti-BAX6A7 co-immunoprecipitates from crude mitochondria lysed in 1% CHAPS were subjected to MS analysis along whole cells as control fraction and isolated crude mitochondria as input fraction. (**b**) List of activated-BAX interactors selected following rigid selection criteria: (i) interactors should be detected only in immunoprecipitates from senescent cells and absent in immunoprecipitates from proliferative cells (ii) interactors should not be increased in the mitochondrial fraction of senescent cells (protein abundance in the input fraction

of senescent cells should be equal to or lower than in proliferative cells) (iii) both unique peptides and calculated protein intensities should equal zero in immunoprecipitates from proliferative cells. As a proof of concept, activated BAX has been only immunoprecipitated from the mitochondria of senescent cells. Identified interactors belonged to several functional groups including mitochondrial dynamics and trafficking, mitochondrial energy metabolism, mitochondrial lipid homeostasis, immunity, and others.

Supplementary Figure 6

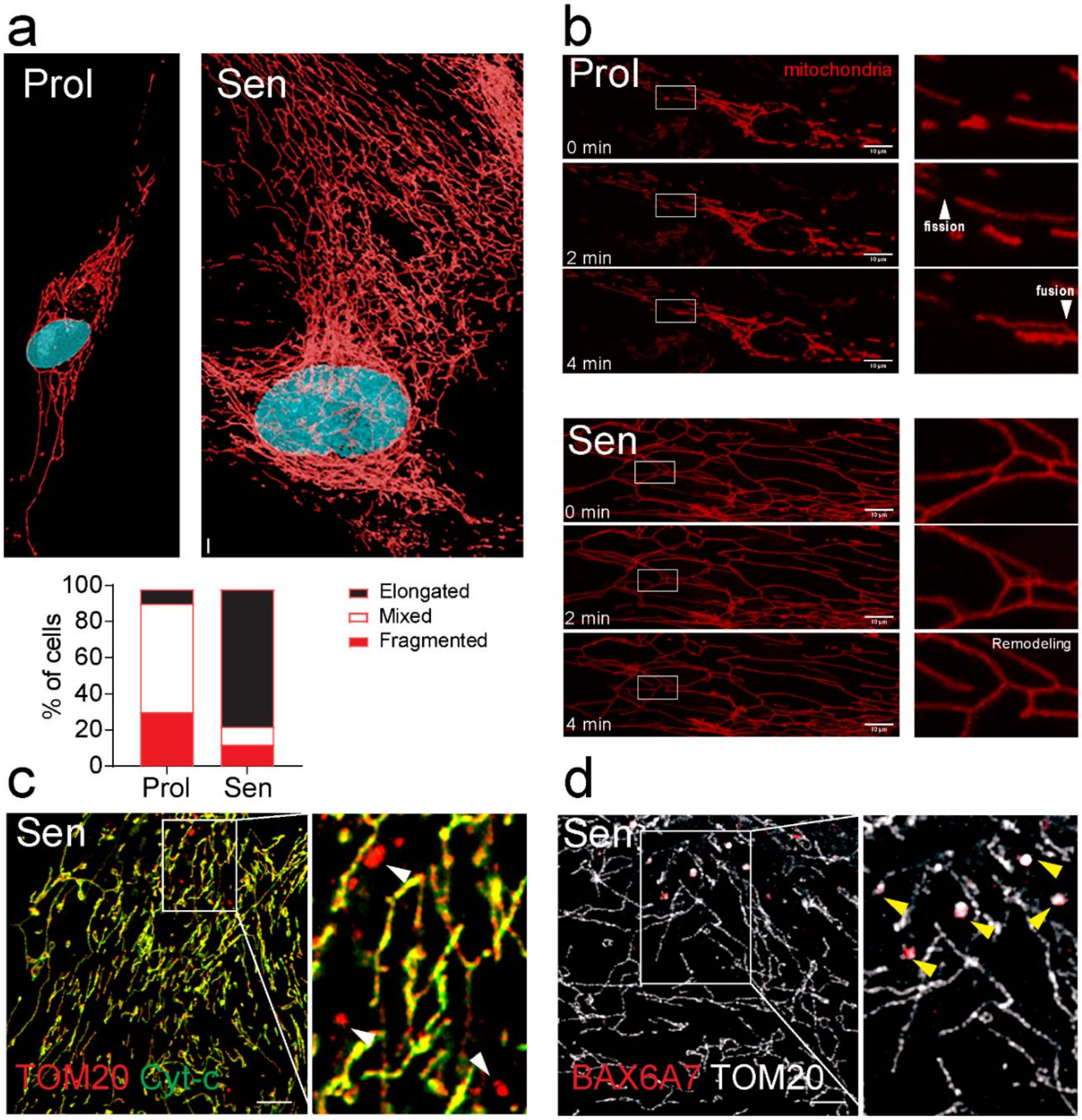

Supplementary Figure 6 – Senescent cells exhibit a hyperfused mitochondrial network and only fragmented mitochondria show miMOMP.

(a) (Top) Representative immunofluorescence image of proliferating and senescent (IR) IMR90 fibroblasts labelled with MitoTracker Red. (Bottom) Quantification of the percentage of proliferating and senescent cells containing elongated, mixed, and fragmented mitochondria network (data from 1 representative experiment) (scale bar is 10µm). **(b)** Representative live-cell image of proliferating and senescent IMR90 human fibroblasts labelled with CellLightTM Mitochondria-RFP at the time points indicated. Magnifications on the right show mitochondria network undergoing fission and fusion events. Representative immunofluorescence images of **(c)**

TOM20 (red) and cytochrome c (green), with magnification showing fragmented mitochondria lacking cytochrome c, and **(d)** BAX6A7 (red) and TOM20 (white) in senescent cells, with magnification showing fragmented mitochondria co-localizing with BAX6A7. Scale bars are 10µm. Images are representative of n=3 independent experiments.

Supplementary Figure 7

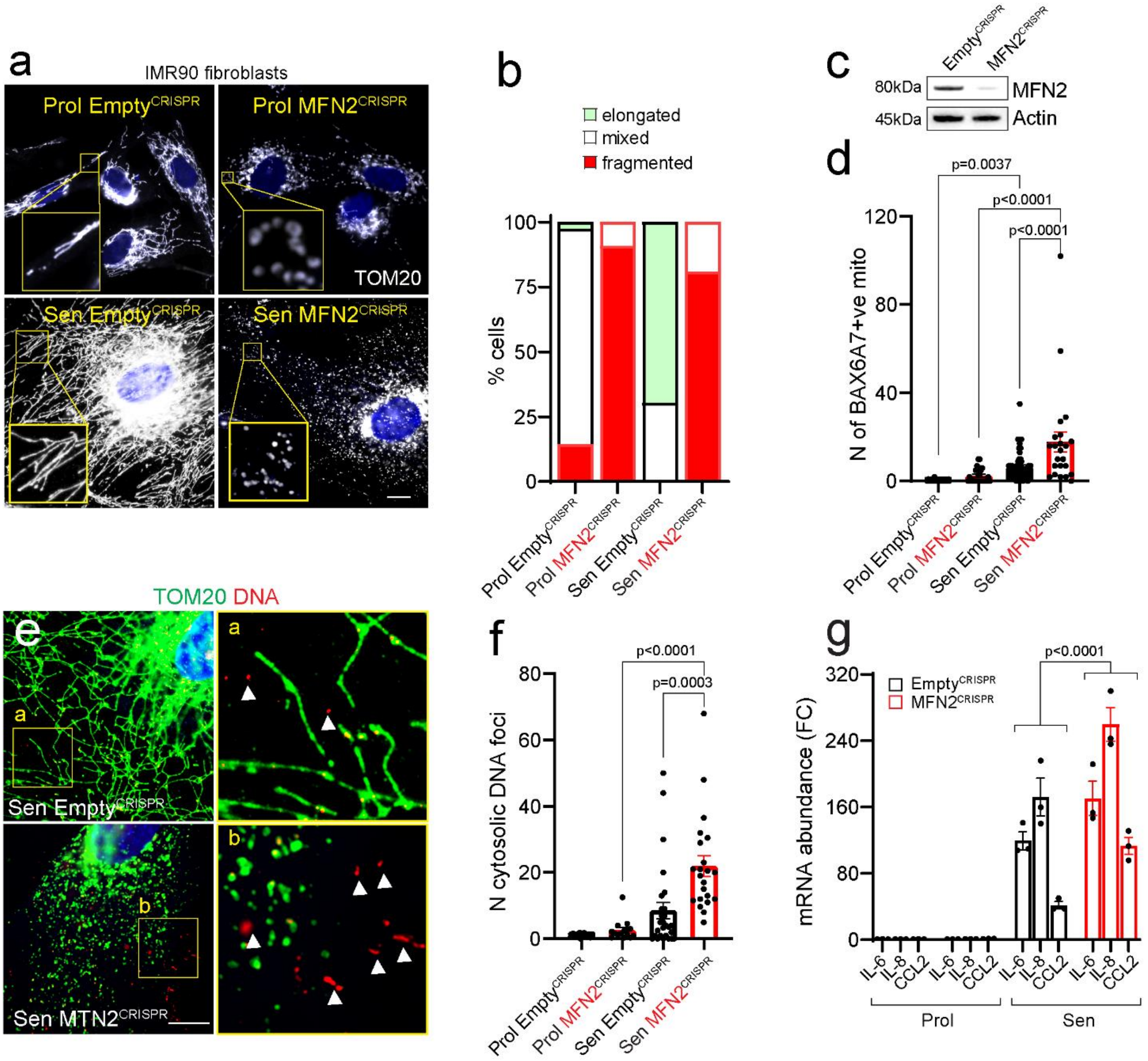

Supplementary Figure 7 – MFN2 deficiency exacerbates mtDNA release and the SASP in senescent IMR90 fibroblasts.

(a) Representative immunofluorescence images of TOM20 (white) in proliferating and senescent (IR) control (EmptyCRISPR) and MFN2-deficient (MFN2CRISPR) IMR90 fibroblasts (scale bar in 20µm). Images are representative of n=2 independent experiments. **(b)** Quantification of the percentage of control and MFN2-deficient IMR90 fibroblasts containing elongated, mixed, and fragmented mitochondrial network (n=2 independent experiments). **(c)** Representative Western blot showing successful CRISPR-Cas9-mediated MFN2 deletion. Quantification of **(d)** the number of BAX6A7-positive mitochondria in proliferating and senescent controls and MFN2-deficient

IMR90 fibroblasts (n=56 and n=27 proliferating control and $MFN2^{-/-}$ cells, respectively; n=49 and n=23 senescent control and $MFN2^{-/-}$ cells, respectively, analyzed over 2 independent experiments). **(e)** Representative immunofluorescence images of TOM20 (green) and DNA (red) in proliferating and senescent control and MFN2-deficient IMR90 fibroblasts (scale bar is 20µm). Magnification on the right shows DNA foci located outside of TOM20. Images are representative of n=2 Independent experiments. **(f)** Quantification of the number of DNA foci found in the cytosol of proliferating and senescent control and MFN2-deficient IMR90 fibroblasts. Each dot represents the average number of DNA foci per cell per image (n=139 and n=96 proliferating control and $MFN2^{-/-}$ cells, respectively; n=50 and n=38 senescent control and $MFN2^{-/-}$ cells, respectively, analyzed over 2 independent experiments. **(g)** Quantification of mRNA expression levels of SASP genes in control and MFN2-deficient IMR90 fibroblasts (n=3 independent experiments). Data are mean ± S.E.M. Statistical significance was assessed using one-way ANOVA followed by Tukey's multiple comparison test (d, f, g). For gel source data (c), see Supplementary Figure 1.

Supplementary Figure 8

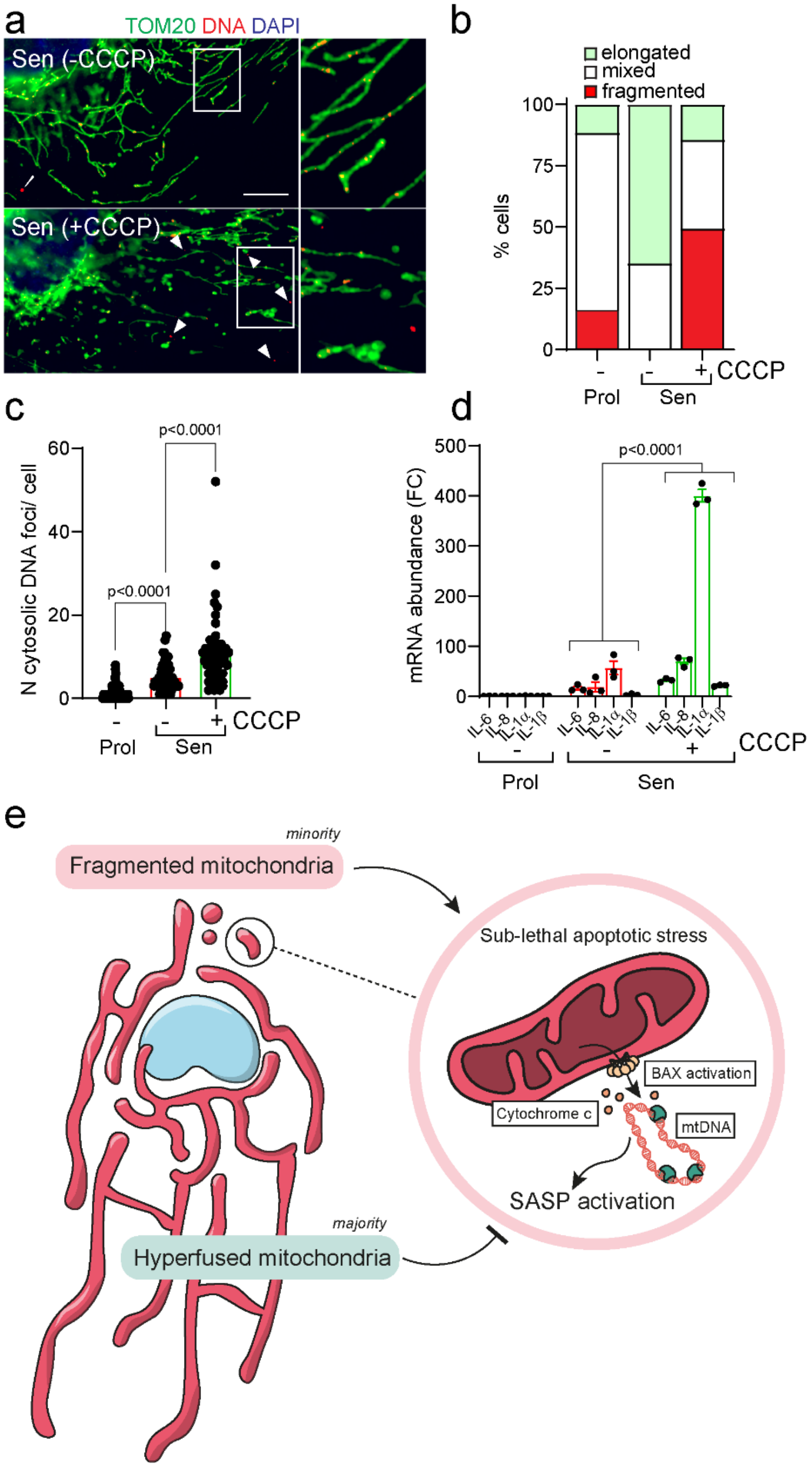

Supplementary Figure 8 – CCCP-induced mitochondrial fragmentation exacerbates intracellular mtDNA release and the SASP during senescence.

(a) Representative immunofluorescence image of TOM20 (green) and DNA (red) in senescent cells with or without CCCP treatment (scale bar is 20µm). Magnification shows DNA foci outside of TOM20. Images are representative of n=3 independent experiments. **(b)** Quantification of the

percentage of cells containing elongated, mixed, and fragmented mitochondrial network following treatment with CCCP (n=111 Prol, n=65 Sen, n=43 Sen + CCCP cells analyzed over 3 independent experiments). **(c)** Quantification of the number of DNA foci found in the cytosol of proliferating and senescent (IR) cells at the conditions indicated (n=92 proliferating cells; n=63 senescent control cells; n=57 senescent cells treated with CCCP analyzed over 3 independent experiments). **(d)** Quantification of mRNA expression levels of the SASP genes indicated in proliferating and senescent cells following CCCP treatment. Data are mean of n=3 independent experiments. **(e)** Scheme depicting our hypothesis: In senescent cells majority of mitochondria can be found in a hyperfused state which limits MOMP and the SASP. Only a small subset of fragmented mitochondria undergoes MOMP (miMOMP) and this is sufficient to account for the increased cytosolic mtDNA, activation of cGAS-STING and the SASP. Data are mean ± S.E.M. Statistical significance was assessed using one-way ANOVA followed by Tukey's multiple comparison test (c), two-way ANOVA followed by Tukey's multiple comparison test (d).

Supplementary Figure 9

Individual and mean brain concentration of BAI1 (ng/g)

Time (h)	M01+3n	M02+3n	M03+3n	Mean	SD
0	BQL*	BQL	BQL	ND*	± ND
1	152	170	176	166	± 12.5
2	159	164	200	174	± 22.4
4	244	284	286	271	± 23.7
8	427	389	389	402	± 21.9
24	805	860	845	837	± 28.4

BQL: = Below the lower limit of quantitation
ND: Not determined

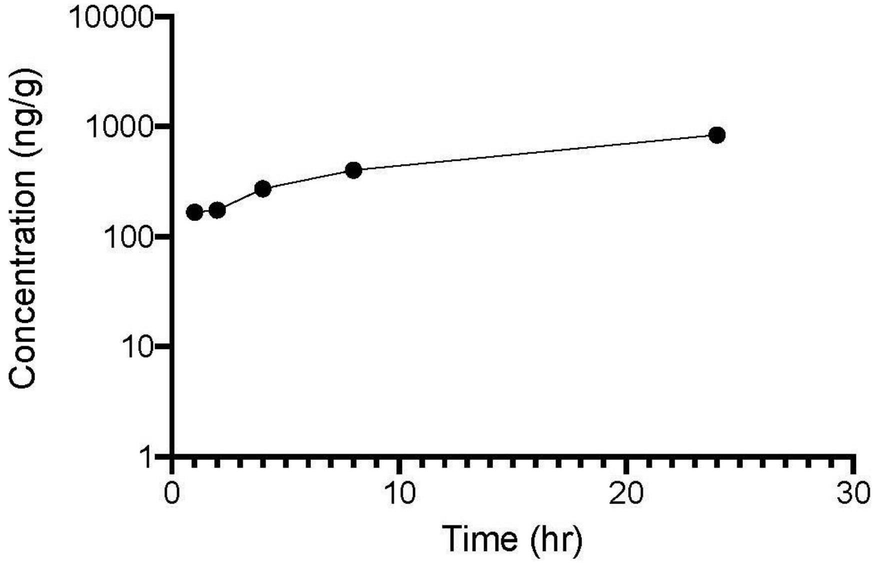

Supplementary Figure 9 – BAI1 crosses the Blood Brain Barrier.
Above- table showing individual and mean concentration of BAI1 detected in brain tissue from aged (>16 months old) C57BL/6J male mice injected with BAI1 at different time points (n=3 mice per time point). Below- graph depicting kinetics of BAI1 in brain tissue up to 24 hours.

Supplemental tables:

Table 1: – Antibodies used for immunocytochemistry and immunohistochemistry

Primary antibodies	Source	Product number	Concentration
Anti-TOMM20 rabbit polyclonal antibody	Millipore Sigma	HPA011562	1:200
Anti-Cytochrome c mouse monoclonal antibody	BioLegend	612301	1:1000
Anti-Bax(6A7) mouse monoclonal antibody	Santa Cruz	sc-23959	1:100
Anti-Phospho-Histone H2A.X (Ser139) (20E3) rabbit monoclonal antibody	Cell Signaling	9718	1:1000
Anti-DNA mouse monoclonal antibody	Millipore Sigma	CBL186	1:100
Anti-TFAM rabbit monoclonal antibody	Cell Signaling	8076S	1:100
Anti-p16 (E6H4) mouse monoclonal antibody	Roche	705-4713	1:5
Anti p21 Waf1 Cip1 (12D1) rabbit monoclonal antibody	Cell Signaling	2947S	1:1000
Anti-Ki67 rabbit polyclonal antibody	Abcam	ab15580	1:1000
Anti-Bax (D3R2M) rabbit monoclonal antibody	Cell Signaling	14796	1:100
Anti-Cleaved Caspase-3 (Asp175) Antibody	Cell Signaling	9661	1:50
**Secondary antibodies**	**Source**	**Product number**	**Concentration**
Goat anti-Rabbit IgG (H+L), Superclonal™ Recombinant Secondary Antibody, Alexa Fluor 647	Thermo Fisher Scientific	A-27040	1:1000
Goat anti-Rabbit IgG (H+L) Cross-Adsorbed Secondary Antibody, Alexa Fluor 488	Thermo Fisher Scientific	A-11008	1:1000
Goat anti-Rabbit IgG (H+L) Cross-Adsorbed Secondary Antibody, Alexa Fluor 594	Thermo Fisher Scientific	A-11012	1:1000
Goat anti-Mouse IgG (H+L) Highly Cross-Adsorbed Secondary Antibody, Alexa Fluor 594	Thermo Fisher Scientific	A-11032	1:1000
Goat anti-Mouse IgG (H+L) Cross-Adsorbed Secondary Antibody, Alexa Fluor 647	Thermo Fisher Scientific	A-21235	1:1000
Goat anti-Mouse IgG (H+L), Superclonal™ Recombinant	Thermo Fisher Scientific	A-28175	1:1000

Secondary Antibody, Alexa Fluor 488			

Table 2: – Antibodies used for Western blotting.

Primary antibodies	Source	Catalog number	Concentration
Anti-cytochrome c (D18C7) rabbit monoclonal antibody	Cell Signaling	11940S	1:1000
Anti-UQCR2 rabbit monoclonal antibody	Abcam	ab103616	1:1000
Anti-Cleaved Caspase 3 (D175 hAIE) rabbit monoclonal antibody	Cell Signaling	9664S	1:500
Anti-β-actin mouse monoclonal antibody	Abcam	ab8226	1:10000
Anti-TFAM rabbit monoclonal antibody	Cell Signaling	8076S	1:200
Anti-Bak (D4E4) rabbit monoclonal antibody	Cell Signaling	12105	1:1000
Anti-Bax (D2E11) rabbit monoclonal antibody	Cell Signaling	5023	1:1000
Anti-Bax (6A7) mouse monoclonal antibody	Santa Cruz	sc-23959	1:1000
Anti-β-Tubulin rabbit polyclonal antibody	Cell Signaling	2146S	1:10000
Anti-Lamin B1 rabbit polyclonal antibody	Abcam	ab16048	1:1000
Anti-HMGB1 rabbit monoclonal	Cell Signaling	6893S	1:1000
Anti-NDUFB8 mouse monoclonal antibody	Abcam	ab110242	1:1000
Anti-GAPDH rabbit monoclonal antibody	Cell Signaling	5174S	1:1000
Anti-cGAS (D1D3G) rabbit monoclonal antibody	Cell Signaling	15102	1:1000
Anti-STING (D2P2F) rabbit monoclonal antibody	Cell Signaling	13647S	1:1000
Anti-APAF1 rabbit monoclonal antibody	Cell Signaling	8723S	1:500
**Secondary antibodies**	**Source**	**Catalog number**	**Concentration**
Goat Anti-Rabbit HRP Conjugated	Sigma Aldrich	A0545	1:5000
Goat Anti-Mouse HRP Conjugated	Sigma Aldrich	A2554	1:5000

Table 3: – qPCR Primer assays used.

Human qPCR Primer Assay	Source	Product number
CDKN1a (p21)	IDT	Hs.PT.58.38492863.g
CDKN2a (p16) (Fig. 3e, Supp 3)	IDT	Hs.PT.58.40743463.g
CDKN2b (Supp. 3)	IDT	Hs.PT.58.4919581
CX3CL1 (Supp. 2f)	IDT	Hs.PT.58.19601997
IFN-α	IDT	Hs.PT.58.39481063.g
IFN-β	IDT	Hs.PT.58.46311748.g
IL-1β (Supp. 2e, Supp. 3)	IDT	Hs.PT.58.1518186
IL-1α	IDT	Hs.PT.58.40913627
IL-6	IDT	Hs.PT.58.40226675
IL-8	IDT	Hs.PT.58.39926886.g
MCP1 (CCL2) (Supp. 3)	IDT	Hs.PT.58.45467977
RSP16	IDT	Hs.PT.58.366374
**Mice qPCR Primer Assay**	**Source**	**Product number**
Bax	IDT	Mm.PT.58.14012210
Ccl2	IDT	Mm.PT.58.42151692
Cdkn1a (p21)	IDT	Mm.PT.58.5884610
Cdkn2a (p16)	IDT	Mm.PT.58.42804808
Col3a1	IDT	Mm.PT.58.13848686
Cx3cl1	IDT	Mm.PT.58.8767901
Cxcl1	IDT	Mm.PT.58.42076891
Cxcl12	IDT	Mm.PT.58.12038563
Cxcl14	IDT	Mm.PT.58.21980826
Ifi44	IDT	Mm.PT.58.12162024
Il1-α	IDT	Mm.PT.58.32778767
Il1-β	IDT	Mm.PT.58.41616450
Il-6	IDT	Mm.PT.58.13354106
Il7	IDT	Mm.PT.58.10325839
Inhibin-a	IDT	Mm.PT.58.11832629
Mmp13	IDT	Mm.PT.58.42286812
Mmp2	IDT	Mm.PT.58.9606100
Mmp3	IDT	Mm.PT.58.9719290
Oas1b	IDT	Mm.PT.56a.10289138.g
Oasl2	IDT	Mm.PT.58.17167264

Table 4: – Sybr Green primers used for qPCR.

Gene name	Forward and reverse PCR primer sequences
hMT-Dloop	CATCTGGTTCCTACTTCAGGG<br>CCGTGAGTGGTTAATAGGGTG
mMT-Dloop	AATCTACCATCCTCCGTGAAACC<br>TCAGTTTAGCTACCCCCAAGTTTAA
mActinb	ATGCTCCCCGGGCTGTAT<br>CATAGGAGTCCTTCTGACCCATTC
mCdkn2a	GAACTCTTTCGGTCGTACCC<br>CGAATCTGCACCGTAGTTGA
mCxcl15	GTCCTTAACCTAGGCATCTTCG<br>TCTGTTGCAGTAAATGGTCTCG
mIl6	TGATGCACTTGCAGAAAACA<br>ACCAGAGGAAATTTTCAATAGGC
mStat1	CGCGCATGCAACTGGCATATAACT<br>ATGCTTCCGTTCCCACGTAGACTT
mIfit3	TTCCCAGCAGCACAGAAAC<br>AAATTCCAGGTGAAATGGCA
mIfi44	CTGATTACAAAAGAAGACATGACAGAC<br>AGGCAAAACCAAAGACTCCA
mIL6	CTACCAAACTGGATATAATCAGGA<br>CCAGGTAGCTATGGTACTCCAGAA
mIL1a	AGGAGAGCCGGGTGACAGTA<br>TCAGAATCTTCCCGTTGCTTG
mIL-1b	CCAAAAGATGAAGGGCTGCT<br>TCATCAGGACAGCCCAGGTC
mCXCL1	CAATGAGCTGCGCTGTCAGT<br>TTGAGGTGAATCCCAGCCAT
mMMP3	TGGAGCTGATGCATAAGCCC<br>TGAAGCCACCAACATCAGGA
mIFNA	GGACTTTGGATTCCCGCAGGAGAAG<br>GCTGCATCAGACAGCCTTGCAGGTC
mOAS1	GCCTGATCCCAGAATCTATGC<br>GAGCAACTCTAGGGCGTACTG
mInhibinA	GATCATCACCTTTGCCGAGT<br>TGGTCCTGGTTCTGTTAGCC
m18s	GTAACCCGTTGAACCCCATT<br>CCATCCAATCGGTAGTAGCG
mCcl2	GTCTGTGCTGACCCCAAGAAG<br>TGGTTCCGATCCAGGTTTTTA
mCcl3	TCCCAGCCAGGTGTCATTTT<br>TTGGAGTCAGCGCAGATCTG
mCcl5	GCCCACGTCAAGGAGTATTTCT<br>ACAAACACGACTGCAAGATTGG
mCcl7	CCCTGGGAAGCTGTTATCTTCA<br>CTGATGGGCTTCAGCACAGA
mCsf1	ATTGCCAAGGAGGTGTCAGAA<br>GGACCTTCAGGTGTCCATTCC

mCxcl1	CCGAAGTCATAGCCACACTCAA<br>CAAGGGAGCTTCAGGGTCAAG
mCxcl2	TCAAGGGCGGTCAAAAAGTT<br>CAGTTAGCCTTGCCTTTGTTCA
mCxcl15	TCCATGGGTGAAGGCTACTGT<br>TTCATTGCCGGTGGAAATTC
mHmgb1	TCCTTCGGCCTTCTTCTTGTT<br>AGGATGCTCGCCTTTGATTTT
mIcam1	GTGGCGGGAAAGTTCCTGTT<br>GTCCAGCCGAGGACCATACA
mIgfbp2	GCCCCCTGGAACATCTCTACT<br>GTTGTACCGGCCATGCTTGT
mIgfbp4	GCAACTTCCACCCCAAACAGT<br>CCTGTCTTCCGATCCACACA
mIl1a	AAGAGACCATCCAACCCAGATC<br>CCTGACGAGCTTCATCAGTTTG
mIl1b	TCAGGCAGGCAGTATCACTCA<br>CACGGGAAAGACACAGGTAGCT
mIl6	ACCACGGCCTTCCCTACTTC<br>TTGGGAGTGGTATCCTCTGTGA
mIl17a	GGACTCTCCACCGCAATGAA<br>GCACTGAGCTTCCCAGATCAC
mInhba	CAGGAAGACACTGCACTTTGA<br>TTCAGGAAGAGCCACACTTCT
mMmp3	TTGACGATGATGAACGATGGA<br>GAGCAGCAACCAGGAATAGGTT
mMmp9	TGAGTCCGGCAGACAATCCT<br>CCCTGGATCTCAGCAATAGCA
mMmp12	GTGCCCGATGTACAGCATCTT<br>GGTACCGCTTCATCCATCTTG
mMmp13	TGAGGAAGACCTTGTGTTTGCA<br>GCAAGAGTCGCAGGATGGTAGT
mNfkb1	GGCTTTGCAAACCTGGGAAT<br>TCCGTGCTTCCAGTGTTTCA
mPappa	CATCTCAGGTGTGTCGAACCA<br>TGCAAGGATACCAAGCATGCT
mSerpine1	GGACACCCTCAGCATGTTCA<br>CGGAGAGGTGCACATCTTTCT
mSerpinb2	TTCCGCATACTGGAAACATCAG<br>GGATGCGTCCTCAATCTCATC
mTnf	GTTCTGCAAAGGGAGAGTGG<br>GCACCTCAGGGAAGAGTCTG
mActb	AATCGTGCGTGACATCAAAGAG<br>GCCATCTCCTGCTCGAAGTC
mHprt	CGTGATTAGCGATGATGAACCA<br>TCCAAATCCTCGGCATAATGA
mTuba1	GGTTCCCAAAGATGTCAATGCT<br>CAAACTGGATGGTACGCTTGGT
