# Supplementary material for: Apoptotic stress causes mtDNA release during senescence and drives the SASP
Source: Nature. 2023 Oct 11;622(7983):627–36. doi: 10.1038/s41586-023-06621-4 (PMC10584674; doi:10.1038/s41586-023-06621-4)
Supplement: Supplementary file 2 — Reporting Summary [file 41586_2023_6621_MOESM2_ESM.pdf]

nature portfolio

Corresponding author(s): Joao Passos

Last updated by author(s): 8/22/2023

Reporting Summary

Nature Portfolio wishes to improve the reproducibility of the work that we publish. This form provides structure for consistency and transparency in reporting. For further information on Nature Portfolio policies, see our Editorial Policies and the Editorial Policy Checklist.

Statistics

For all statistical analyses, confirm that the following items are present in the figure legend, table legend, main text, or Methods section.

n/a	Confirmed	
☐	☒	The exact sample size (*n*) for each experimental group/condition, given as a discrete number and unit of measurement
☐	☒	A statement on whether measurements were taken from distinct samples or whether the same sample was measured repeatedly
☐	☒	The statistical test(s) used AND whether they are one- or two-sided<br>*Only common tests should be described solely by name; describe more complex techniques in the Methods section.*
☐	☒	A description of all covariates tested
☐	☒	A description of any assumptions or corrections, such as tests of normality and adjustment for multiple comparisons
☐	☒	A full description of the statistical parameters including central tendency (e.g. means) or other basic estimates (e.g. regression coefficient) AND variation (e.g. standard deviation) or associated estimates of uncertainty (e.g. confidence intervals)
☐	☒	For null hypothesis testing, the test statistic (e.g. *F*, *t*, *r*) with confidence intervals, effect sizes, degrees of freedom and *P* value noted<br>*Give P values as exact values whenever suitable.*
☒	☐	For Bayesian analysis, information on the choice of priors and Markov chain Monte Carlo settings
☒	☐	For hierarchical and complex designs, identification of the appropriate level for tests and full reporting of outcomes
☒	☐	Estimates of effect sizes (e.g. Cohen's *d*, Pearson's *r*), indicating how they were calculated

Our web collection on statistics for biologists contains articles on many of the points above.

Software and code

Policy information about availability of computer code

Data collection	No software was used
Data analysis	GraphPad Prism v9

For manuscripts utilizing custom algorithms or software that are central to the research but not yet described in published literature, software must be made available to editors and reviewers. We strongly encourage code deposition in a community repository (e.g. GitHub). See the Nature Portfolio guidelines for submitting code & software for further information.

Data

Policy information about availability of data

All manuscripts must include a data availability statement. This statement should provide the following information, where applicable:

- Accession codes, unique identifiers, or web links for publicly available datasets
- A description of any restrictions on data availability
- For clinical datasets or third party data, please ensure that the statement adheres to our policy

The RNA-seq datasets generated and analyzed during the current study are available in the GEO repository GSE196610 and GSE235225. The mass spectrometry proteomics data have been deposited to the ProteomeXchange Consortium via the PRIDE partner repository with the dataset identifier PXD040018.

Field-specific reporting

Please select the one below that is the best fit for your research. If you are not sure, read the appropriate sections before making your selection.

☒ Life sciences ☐ Behavioural & social sciences ☐ Ecological, evolutionary & environmental sciences

For a reference copy of the document with all sections, see nature.com/documents/nr-reporting-summary-flat.pdf

Life sciences study design

All studies must disclose on these points even when the disclosure is negative.

Sample size	For in vivo mouse studies, we have projected group size estimates based on power analyses and previous experiences.
Data exclusions	no data was excluded
Replication	we tested our hypotheses using multiple approaches when feasible to enhance scientific rigor and reproducibility.<br>in vitro all experiments were performed at least 3 times independently.
Randomization	Animals were randomly assigned numbers at weaning. Once assigned to groups, the genotype was not be linked to the numbers until data analysis following completion of all studies.
Blinding	Investigators were blinded to allocation during experiments and outcome assessments, and data was collected and analyzed in a blinded fashion.

Reporting for specific materials, systems and methods

We require information from authors about some types of materials, experimental systems and methods used in many studies. Here, indicate whether each material, system or method listed is relevant to your study. If you are not sure if a list item applies to your research, read the appropriate section before selecting a response.

Materials & experimental systems

n/a	Involved in the study	
☐	☒	Antibodies
☐	☒	Eukaryotic cell lines
☒	☐	Palaeontology and archaeology
☐	☒	Animals and other organisms
☒	☐	Human research participants
☒	☐	Clinical data
☒	☐	Dual use research of concern

Methods

n/a	Involved in the study	
☒	☐	ChIP-seq
☒	☐	Flow cytometry
☒	☐	MRI-based neuroimaging

Antibodies

Antibodies used

Anti-TOMM20 rabbit polyclonal antibody; Millipore Sigma; HPA011562
Anti-Cytochrome c mouse monoclonal antibody; BioLegend; 612301
Anti-Bax(6A7) mouse monoclonal antibody; Santa Cruz; sc-23959
Anti-Phospho-Histone H2A.X (Ser139) (20E3) rabbit monoclonal antibody; Cell Signaling; 9718
Anti-DNA mouse monoclonal antibody; Millipore Sigma; CBL186
Anti-TFAM rabbit monoclonal antibody; Cell Signaling; 8076S
Anti-p16 (E6H4) mouse monoclonal antibody; Roche; 705-4713
Anti p21 Waf1 Cip1 (12D1) rabbit monoclonal antibody; Cell Signaling; 2947S
Anti-Ki67 rabbit polyclonal antibody; Abcam; ab15580
Anti-Bax (D3R2M) rabbit monoclonal antibody; Cell Signaling; 14796
Anti-Cleaved Caspase-3 (Asp175) Antibody; Cell Signaling; 9661
Goat anti-Rabbit IgG (H+L), Superclonal™ Recombinant Secondary Antibody, Alexa Fluor 647; Thermo Fisher Scientific; A-27040
Goat anti-Rabbit IgG (H+L) Cross-Adsorbed Secondary Antibody, Alexa Fluor 488; Thermo Fisher Scientific; A-11008
Goat anti-Rabbit IgG (H+L) Cross-Adsorbed Secondary Antibody, Alexa Fluor 594; Thermo Fisher Scientific; A-11012
Goat anti-Mouse IgG (H+L) Highly Cross-Adsorbed Secondary Antibody, Alexa Fluor 594; Thermo Fisher Scientific; A-11032
Goat anti-Mouse IgG (H+L) Cross-Adsorbed Secondary Antibody, Alexa Fluor 647; Thermo Fisher Scientific; A-21235
Goat anti-Mouse IgG (H+L), Superclonal™ Recombinant Secondary Antibody, Alexa Fluor 488; Thermo Fisher Scientific; A-28175
Anti-cytochrome c (D18C7) rabbit monoclonal antibody; Cell Signaling; 11940S
Anti-UQCR2 rabbit monoclonal antibody; Abcam; ab103616
Anti-Cleaved Caspase 3 (D175 hAIE) rabbit monoclonal antibody; Cell Signaling; 9664S
Anti-β-actin mouse monoclonal antibody; Abcam; ab8226
Anti-TFAM rabbit monoclonal antibody; Cell Signaling; 8076S

Anti-Bak (D4E4) rabbit monoclonal antibody; Cell Signaling; 12105
Anti-Bax (D2E11) rabbit monoclonal antibody; Cell Signaling; 5023
Anti-Bax (6A7) mouse monoclonal antibody; Santa Cruz; sc-23959
Anti-[illegible]-Tubulin rabbit polyclonal antibody; Cell Signaling; 2146S
Anti-Lamin B1 rabbit polyclonal antibody; Abcam; ab16048
Anti-NDUFB8 mouse monoclonal antibody; Abcam; ab110242
Anti-GAPDH rabbit monoclonal antibody; Cell Signaling; 5174S
Anti-cGAS (D1D3G) rabbit monoclonal antibody; Cell Signaling; 15102
Anti-STING (D2P2F) rabbit monoclonal antibody; Cell Signaling; 13647S
Anti-APAF1 rabbit monoclonal antibody; Cell Signaling; 8723S
Anti-HMGB1 rabbit monoclonal antibody; Cell Signaling; 6893S
Goat Anti-Rabbit HRP Conjugated; Sigma Aldrich; A0545
Goat Anti-Mouse HRP Conjugated; Sigma Aldrich; A2554

Validation

All antibodies used were purchased from commercial sources and reputable vendors (eg. Abcam, Cell Signaling). We selected them based on cross-reactivity with mouse and human (depending on our research needs) and carefully evaluated the validation data provided by both the vendors and literature. In several instances, we performed further validation of antibodies by examining the molecular weight of the band by Western blotting and Knocking out protein of interest. Antibodies were aliquoted and stored as recommended by the manufacturer to minimize freeze thaw cycles and associated loss in performance.

Eukaryotic cell lines

Policy information about cell lines

Cell line source(s)

IMR90 and MRC5 human fibroblasts were acquired from ATCC

Authentication

none of the cells have been authenticated

Mycoplasma contamination

all cell lines used have been regularly tested for Mycoplasm

Commonly misidentified lines
(See ICLAC register)

N/A

Animals and other organisms

Policy information about studies involving animals; ARRIVE guidelines recommended for reporting animal research

Laboratory animals

Information regarding experiments involving laboratory mice is in methods section.

Wild animals

N/A

Field-collected samples

N/A

Ethics oversight

All animal experiments were performed according to protocols approved by the Institutional Animal Care and Use Committee (IACUC) at Mayo Clinic.

Note that full information on the approval of the study protocol must also be provided in the manuscript.
